# POLLAR: Impact of air POLLution on Asthma and Rhinitis; a European Institute of Innovation and Technology Health (EIT Health) project

**DOI:** 10.1186/s13601-018-0221-z

**Published:** 2018-09-17

**Authors:** Jean Bousquet, Josep M. Anto, Isabella Annesi-Maesano, Toni Dedeu, Eve Dupas, Jean-Louis Pépin, Landry Stephane Zeng Eyindanga, Sylvie Arnavielhe, Julia Ayache, Xavier Basagana, Samuel Benveniste, Nuria Calves Venturos, Hing Kin Chan, Mehdi Cheraitia, Yves Dauvilliers, Judith Garcia-Aymerich, Ingrid Jullian-Desayes, Chitra Dinesh, Daniel Laune, Jade Lu Dac, Ismael Nujurally, Giovanni Pau, Robert Picard, Xavier Rodo, Renaud Tamisier, Michael Bewick, Nils E. Billo, Wienczyslawa Czarlewski, Joao Fonseca, Ludger Klimek, Oliver Pfaar, Jean-Marc Bourez

**Affiliations:** 1 MACVIA-France, Fondation partenariale FMC VIA-LR, Montpellier, France; 2 INSERM U 1168, VIMA : Ageing and Chronic Diseases Epidemiological and Public Health Approaches, Villejuif, France; 3 Université Versailles St-Quentin-en-Yvelines, UMR-S 1168, Montigny le Bretonneux, France; 4 Euforea, Brussels, Belgium; 5 Charité, Berlin, Germany; 6 CHU Montpellier, 371 Avenue du Doyen Gaston Giraud, 34295 Montpellier Cedex 5, France; 7 ISGlobal, Centre for Research in Environmental Epidemiology (CREAL), Barcelona, Spain; 8 IMIM (Hospital del Mar Research Institute), Barcelona, Spain; 9 Universitat Pompeu Fabra (UPF), Barcelona, Spain; 10 CIBER Epidemiología y Salud Pública (CIBERESP), Barcelona, Spain; 11 Epidemiology of Allergic and Respiratory Diseases, Department Institute Pierre Louis of Epidemiology and Public Health, INSERM and UPMC Sorbonne Universités, Medical School Saint Antoine, Paris, France; 12 AQuAS, Barcelona, Spain; 13 Kyomed INNOV, Montpellier, France; 14 Université Grenoble Alpes, Laboratoire HP2, INSERM, U1042 Grenoble, France; 15 CHU de Grenoble, Grenoble, France; 16 Bull SAS, Échirolles, France; 17 National Center of Expertise in Cognitive Stimulation (CEN STIMCO), Broca Hospital, Paris, France; 18 Memory and Cognition Laboratory, Institute of Psychology, Paris Descartes University, Sorbonne Paris Cité, Boulogne Billancourt, France; 19 Mines ParisTech CRI - PSL Research University, Fontainebleau, France; 20 Direction de la Recherche, Innovation et Valorisation, Université Grenoble Alpes, Grenoble, France; 21 Neogia, Paris, France; 22 Centre National de Référence Narcolepsie Hypersomnies, Département de Neurologie, Hôpital Gui-de-Chauliac Inserm U1061, Unité des Troubles du Sommeil, Montpellier, France; 23 LIP6 SU, Place Jussieu, Paris, France; 24 Conseil Général de l’Economie Ministère de l’Economie, de l’Industrie et du Numérique, Paris, France; 25 Climate and Health Program and ISGlobal and ICREA, Barcelona, Spain; 26 iQ4U Consultants Ltd, London, UK; 27 Joensuu, Finland; 28 Medical Consulting Czarlewski, Levallois, France; 29 Center for Health Technology and Services Research- CINTESIS, Faculdade de Medicina, Universidade do Porto, Porto, Portugal; 30 MEDIDA, Lda, Porto, Portugal; 31 Center for Rhinology and Allergology, Wiesbaden, Germany; 32 Department of Otorhinolaryngology, Head and Neck Surgery, Universitätsmedizin Mannheim, Medical Faculty Mannheim, Heidelberg University, Mannheim, Germany; 33 Managing Director, EIT Health France, Paris, France

**Keywords:** Asthma, Pollen, Pollution, Rhinitis, mHealth, Climate change

## Abstract

Allergic rhinitis (AR) is impacted by allergens and air pollution but interactions between air pollution, sleep and allergic diseases are insufficiently understood. POLLAR (Impact of air POLLution on sleep, Asthma and Rhinitis) is a project of the European Institute of Innovation and Technology (EIT Health). It will use a freely-existing application for AR monitoring that has been tested in 23 countries (the *Allergy Diary*, iOS and Android, 17,000 users, TLR8). The Allergy Diary will be combined with a new tool allowing queries on allergen, pollen (TLR2), sleep quality and disorders (TRL2) as well as existing longitudinal and geolocalized pollution data. Machine learning will be used to assess the relationship between air pollution, sleep and AR comparing polluted and non-polluted areas in 6 EU countries. Data generated in 2018 will be confirmed in 2019 and extended by the individual prospective assessment of pollution (portable sensor, TLR7) in AR. Sleep apnea patients will be used as a demonstrator of sleep disorder that can be modulated in terms of symptoms and severity by air pollution and AR. The geographic information system GIS will map the results. Consequences on quality of life (EQ-5D), asthma, school, work and sleep will be monitored and disseminated towards the population. The impacts of POLLAR will be (1) to propose novel care pathways integrating pollution, sleep and patients’ literacy, (2) to study sleep consequences of pollution and its impact on frequent chronic diseases, (3) to improve work productivity, (4) to propose the basis for a sentinel network at the EU level for pollution and allergy, (5) to assess the societal implications of the interaction. MASK paper N°32.

## Background

Exposure to ambient air pollution increases morbidity and mortality. It is a leading contributor to global disease burden [1, 2]. The role of air pollution on cardiovascular events [3], COPD [4], sleep apnea [5] and asthma exacerbations [6] is clear. In allergic rhinitis (AR), air pollution is one of the risk factors that induces allergic sensitization and deteriorates the AR condition, but data are sometimes conflicting [7]. Moreover, data on the impact of air pollution on AR multimorbidity [8] or severity are scarce [9] and not always conclusive, probably due to methodological problems.

Meteorological factors such as temperature, sunlight and humidity as well as air pollution can affect pollen emission and allergenic concentration [10–12]. Traffic-related pollutants [13] and diesel exhaust particles can disrupt pollen, leading to the release of pauci-micronic particles which can penetrate in the bronchi [14]. Asthma due to pollen may be associated to peaks of air pollution [15–19]. These data suggest an important interaction between pollens and pollution, inducing asthma in AR patients during the pollen season. However, more data should be collected and mobile technology may be interesting.

MASK-rhinitis (Mobile Airways Sentinel NetworK for allergic rhinitis) is a patient centred ICT system [20]. A mobile phone app (*Allergy Diary)* central to MASK has been launched in 23 countries and has been validated [21–24].

Many different methods are used to monitor pollen exposure [25–28]. Pollen counts can assess the exposure of pollen-allergic patients [29]. The assessment of allergen content in the air is feasible [30] but requires sophisticated methods that may not account for all of the pollen species in the ambient air. Meteorological data may, in the future, be of interest for predicting the onset of the season, but more data are required [31]. Combining several sources using advanced data engineering may also be important but these data are still complex and, in many different areas, not yet available for all pollen species [25–28, 32]. Google Trends (GT) is a Web-based surveillance tool that uses Google to explore the searching trends of specific queries. Recent studies have suggested the utility of GT for assessing the seasonality of allergic diseases [33–37]. GT reflects the real-world AR epidemiology and could potentially be used as a monitoring tool for allergic rhinitis [38, 39].

Interactions between air pollution, sleep quality, sleep disorders [40] and allergic diseases are clear but insufficiently understood. POLLAR (Impact of Air POLLution on sleep, Asthma and Rhinitis) is a new project of the EIT Health that will embed environmental data into the *Allergy Diary*. POLLAR aims at combining emerging technologies (search engine Technology Readiness level TLR2; sleep assessment, pollution sampler TLR6, *Allergy Diary* TLR9) with machine learning to (1) understand the effects of air pollution in allergic rhinitis and its impact on sleep, work and asthma, (2) assess societal consequences, shared with citizens, corporate citizens and professionals (3) propose preventive strategies and (4) develop participative policies.

## EIT health

### European Institute of Innovation and Technology (EIT) and Knowledge and Innovation Communities (KICs)

*The European Institute of Innovation and Technology (EIT)*, the research and technological agency of the EU, was set up in 2008. It aims to spur innovation and entrepreneurship across Europe in order to overcome some of its greatest challenges. The EIT strengthens cooperation among its partners to form dynamic pan-European partnerships and to develop favorable environments for creative thought processes and innovations. Real sustainable products, services, entrepreneurs, engineers, scientists, companies, revenue, profit and jobs are emerging from the Innovation Communities making this innovation network the largest in Europe, if not in the world.

*The Knowledge and Innovation Communities (KICs)* represent a unique feature of the EIT for the integration of education, research and innovation (the so-called *Knowledge Triangle*) in a common organization. The KICs carry out activities that cover the entire innovation chain: training and education programmes, reinforcing the journey from research to the market, innovation projects, as well as business incubators and accelerators.

There are currently six Innovation Communities and each one focuses on a different societal challenge (https://eit.europa.eu/activities/innovation-communities): EIT Climate-KIC (climate change mitigation and adaptation), EIT Digital (Information and Communication Technologies), EIT InnoEnergy (sustainable energy), EIT Health (healthy living and active ageing), EIT Raw Materials (sustainable exploration, extraction, processing, recycling and substitution) and EIT Food (putting Europe at the centre of a global revolution in food innovation and production).

### EIT health

EIT Health (European Institute of Innovation and Technology-Health) is a consortium of over 50 core partners and 90 associate partners from leading businesses, research centres and universities across 14 EU countries. EIT Health works to give EU citizens greater opportunities to enjoy a healthier and active life for longer, and to postpone dependency on others, by leveraging big data and new technologies, identifying and removing barriers to innovation, and building on education and talent creation (https://www.eithealth.eu). EIT Health allows:

- Innovative products and services to be developed in every area imaginable, including climate change, healthy living and active and healthy ageing (AHA).
- New companies to be started.
- A new generation of entrepreneurs to be trained.

The EIT’s role is to guide the process and set the strategies, but the KICs should put these into practice and provide results.

Three pillars have been defined:

- Promote healthy living, self-management of health and life style interventions.
- Support active ageing.
- Improve healthcare with innovations and a patient-centric approach, in particular for chronic diseases.

EIT Health brings together the three sides of the knowledge triangle through three programmes:

- Campus (education) provides up-to-date knowledge, skills and attitudes to help turn the brightest learners into healthcare leaders and entrepreneurs to shape the future of Europe’s health. Campus educational offerings are intended to increase industry knowledge and deliver novel skills, as EIT seeks to inject an entrepreneurial approach into European healthcare education (https://www.eithealth.eu/campus).
- Accelerator (business creation) supports the best and brightest health industry entrepreneurs, creating a favorable environment for innovation and providing skills and services to get promising business ideas into the market.
- Innovation Projects provide comprehensive support for innovations that show the potential to have a positive impact on healthcare for a societal challenge. The most promising ideas are developed into commercially-viable products through a multi-disciplinary approach.

## The Allergy Diary

### MASK (Mobile Airways Sentinel NetworK)

**Fig. 1:**
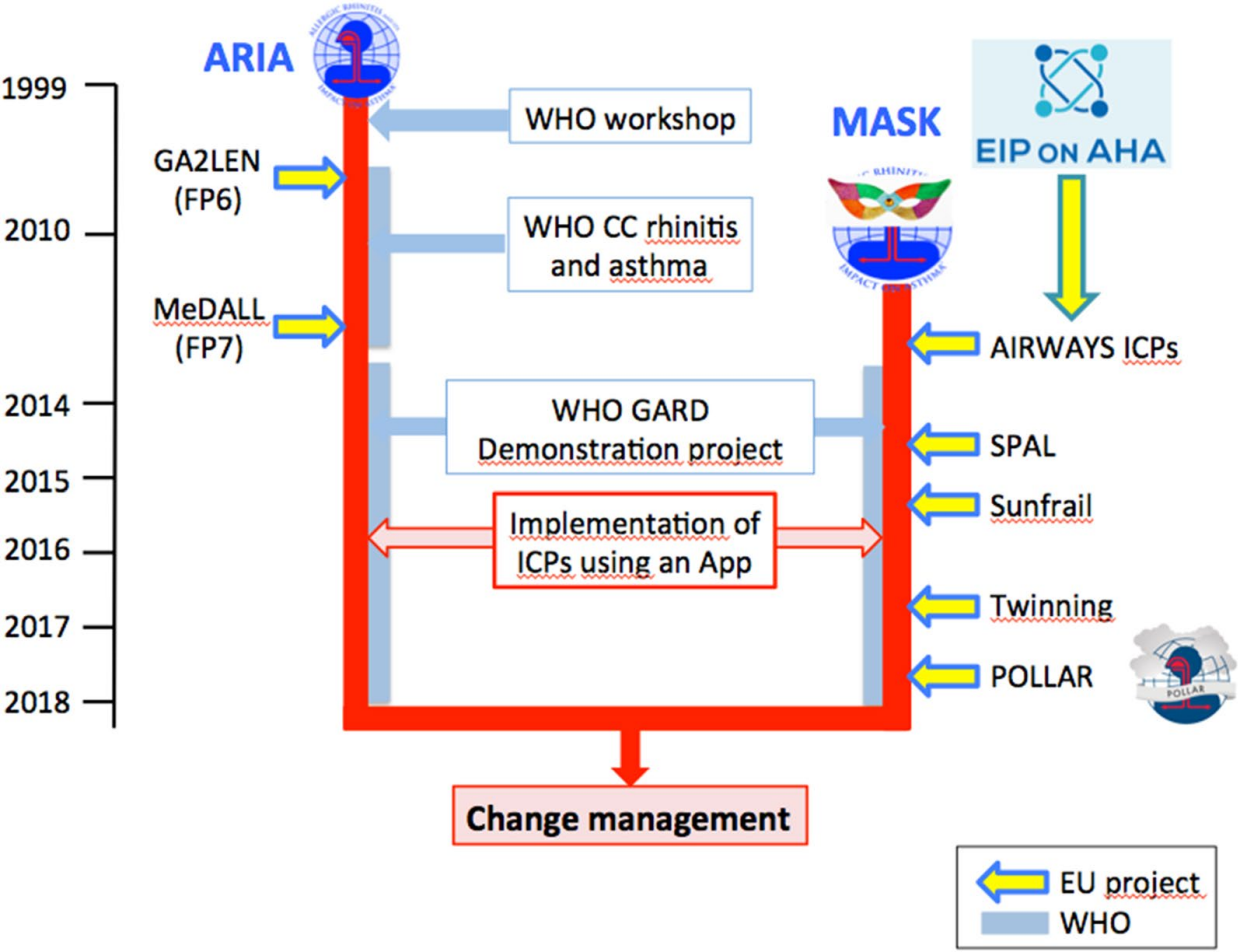
Links between ARIA and MASK for change management. *CC* Collaborating Center, *GA*^*2*^*LEN* Global Allergy and Asthma European network, *GARD* Global Alliance against Chronic Respiratory Diseases, *MeDALL* Mechanisms of the Development of ALLergy, *POLLAR* Impact of air POLLution on sleep, Asthma and Rhinitis

In 2012, the European Commission launched the European Innovation Partnership on Active and Healthy Ageing (DG Santé and DG CONNECT) [41]. The B3 Action Plan devoted to innovative integrated care models for chronic diseases has selected Integrated care pathways for airway diseases (AIRWAYS ICPs) [42, 43] with a life cycle approach [44] as the model for chronic diseases. The Action Plan of AIRWAYS ICPs has been devised [42], implemented [45] and scaled up [46, 47]. AIRWAYS ICPs is a GARD (WHO Global Alliance for Chronic Respiratory Diseases) [48] research demonstration project (Fig. 1).

MASK (Mobile Airways Sentinel NetworK) represents Phase 3 of ARIA and is an AIRWAYS ICPs tool [20, 49]. It represents a Good Practice focusing on the implementation of multi-sectoral care pathways using emerging technologies with real life data in rhinitis and asthma multi-morbidity. MASK follows the JA-CHRODIS (Joint Action on Chronic Diseases and Promoting Healthy Ageing across the Life Cycle, 2nd EU Health Programme 2008-2013 [50]) recommendations for good practices [51].

MASK was initiated to reduce the global burden of rhinitis and asthma, by giving the patient a simple tool to better prevent and manage respiratory allergic diseases. More specifically, MASK should help to (i) understand the disease mechanisms and the effects of air pollution in allergic diseases, (ii) better appraise the burden incurred by medical needs but also the indirect costs, (iii) propose novel multidisciplinary care pathways integrating pollution and patients’ literacy, (iv) improve work productivity, (v) propose the basis for a sentinel network at the EU level for pollution and allergy and (vi) assess the societal implications of the project to reduce health and social inequalities globally.

### The *Allergy Diary*

The mobile technology of MASK is the *Allergy Diary*, an App (Android and iOS) which is freely available for AR and asthma sufferers in 23 countries (16 EU countries, Argentina, Australia, Brazil, Canada, Mexico, Switzerland and Turkey) and 17 languages (translated and back-translated, culturally adapted and legally compliant) [20]. Users fill in a simple questionnaire on asthma and rhinitis upon registration and daily assess the impact of their disease using a visual analogue scale (VAS) [52] for global allergy symptoms, rhinitis, conjunctivitis, asthma and for work. Moreover, two specific questionnaires are applied every week to assess disease impact on patients’ QoL (EQ-5D) [24] and productivity at work (WPAI-AS) [53]. The *Allergy Diary* is associated with an inter-operable tablet with a CDSS for physicians and other health care professionals [54].

Pilot studies in up to 17,000 users and over 95,000 days are available. The *Allergy Diary* has been validated [23] and has shown that (1) totally anonymized geolocation can be used in 22 countries (in preparation), (2) the *Allergy Diary* data can be analyzed in 22 countries and 16 languages, (3) sleep, work productivity and daily activities are impaired in AR [22, 24], (4) daily work productivity is associated with AR severity [21], (5) everyday use of medications can be monitored proposing a novel assessement of treatment patterns (in press), (6) novel patterns of multimorbidity have been identified [55] and confirmed in epidemiological studies [56, 57] and (7) over 80% of AR patients self-medicate and are non-observant (Menditto, in preparation).

The *Allergy Diary* (TLR 9, Technology Readiness level) represents a validated mHealth tool for the management of AR. Asthma has also been monitored but data have not yet been analysed. Economic impact can be monitored using work productivity. The results of the Allergy Diary have made innovative approaches of AR possible and are directly strengthening the Change Management (CM) strategies in ARIA.

### Transfer of innovation of MASK

A Transfer of Innovation (Twinning) project has been funded by the European Innovation Partnership on Active and Healthy Ageing using MASK in 22 Reference Sites or regions across Europe, Australia, Brazil and Mexico [58]. This will improve the understanding, assessment of burden, diagnosis and management of rhinitis in old age by comparison with an adult population. Specific objectives are: (1) to assess the percentage of adults and elderly who are able to use the Allergy Diary, (2) to study phenotypic characteristics and treatment over a period of one year of rhinitis and asthma multimorbidity at baseline (cross-sectional study) (3) to provide some insight into the differences between elderly people and adults in terms of response to treatment and practice.

The Twinning has been tested in Germany (Region Kohl-Bohn) in a pilot study that has now been extended to the other countries of the Twinning project.

## Pollar

### Goals

AR and asthma impact the social life, school and work [59] of dozens of millions of EU citizens [21]. Their impact on work productivity is estimated to cost 30–50 billion € per year in the EU. AR affects sleep quality and the severity of sleep disorders, namely sleep apnea, and is associated with asthma. AR and asthma induce health and social inequalities across the life cycle. Air pollution has a significant impact on AR severity and its consequences. The cost of inaction is unacceptable.

*POLLAR’s mission* is to better understand the links between AR, asthma allergen exposure, sleep and pollution in order to provide preventive and treatment strategies to reduce the burden of AR and asthma.

*POLLAR’s ambition* is to deliver an integrated solution tailored to the needs of EU citizens, employers and healthcare systems (including insurance companies).

*POLLAR’s objective* is to better manage health societal consequences of the disease by providing assistance during peaks of allergens and air pollution.

POLLAR is user-designed with specific functionalities adapted to patients, employers, policy makers and clinicians.

*POLLAR’s aims* are (1) to deliver a medical device/treatment with high eligibility for the stratification of patients who need to be treated with OTC drugs, prescribed drugs or allergen immunotherapy, (2) to provide a sentinel for air pollution and allergen exposure for municipalities or regions that can be relayed by media or social networks, (3) to help reimbursement strategies by health care systems or insurances, (4) to improve work productivity in the workplace, (5) to better understand the reciprocal links between AR, pollution and sleep/sleep disorders and (6) in the end, to reduce health and social inequalities between and within countries.

### Consortium

**Fig. 2:**
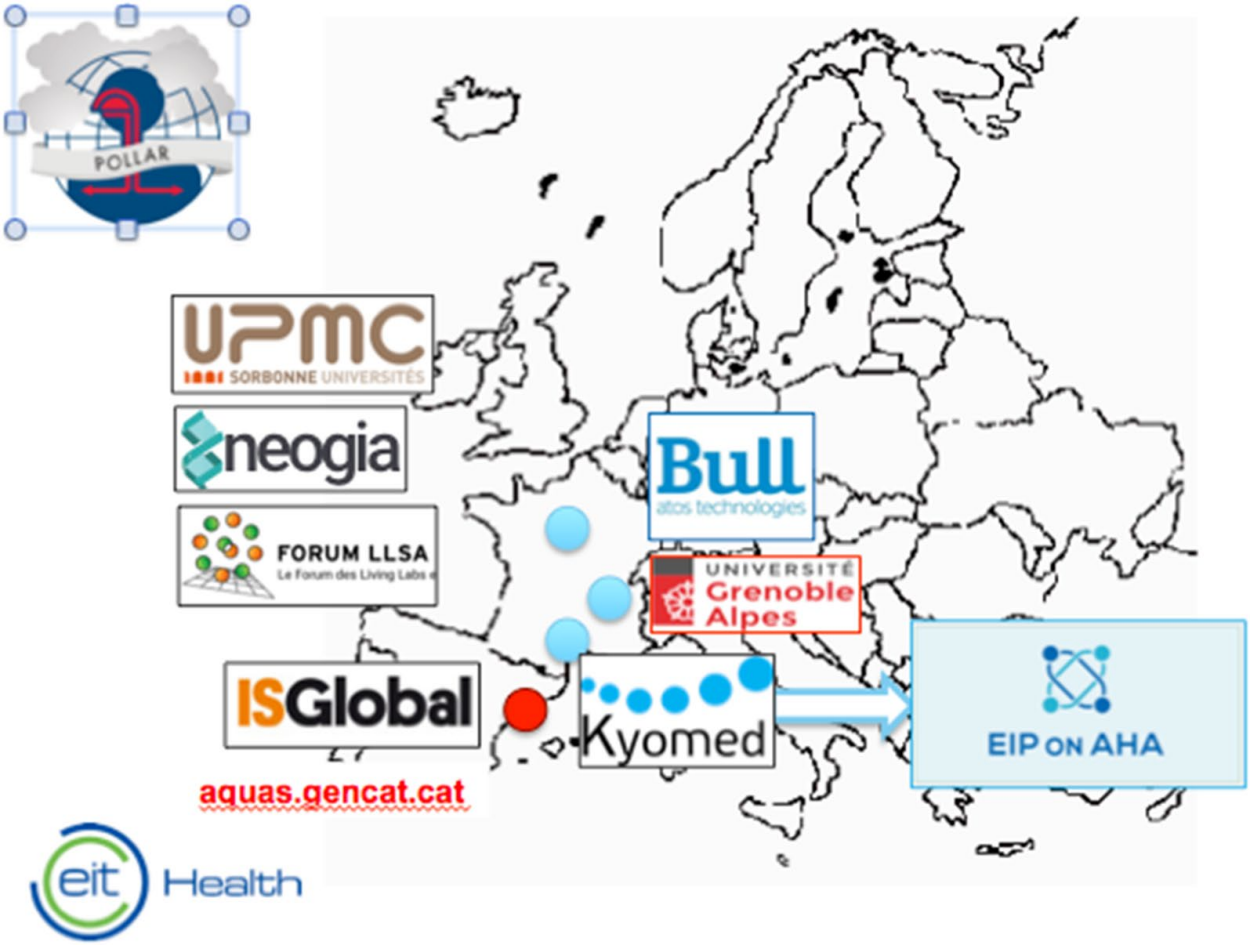
The POLLAR consortium

**Fig. 3:**
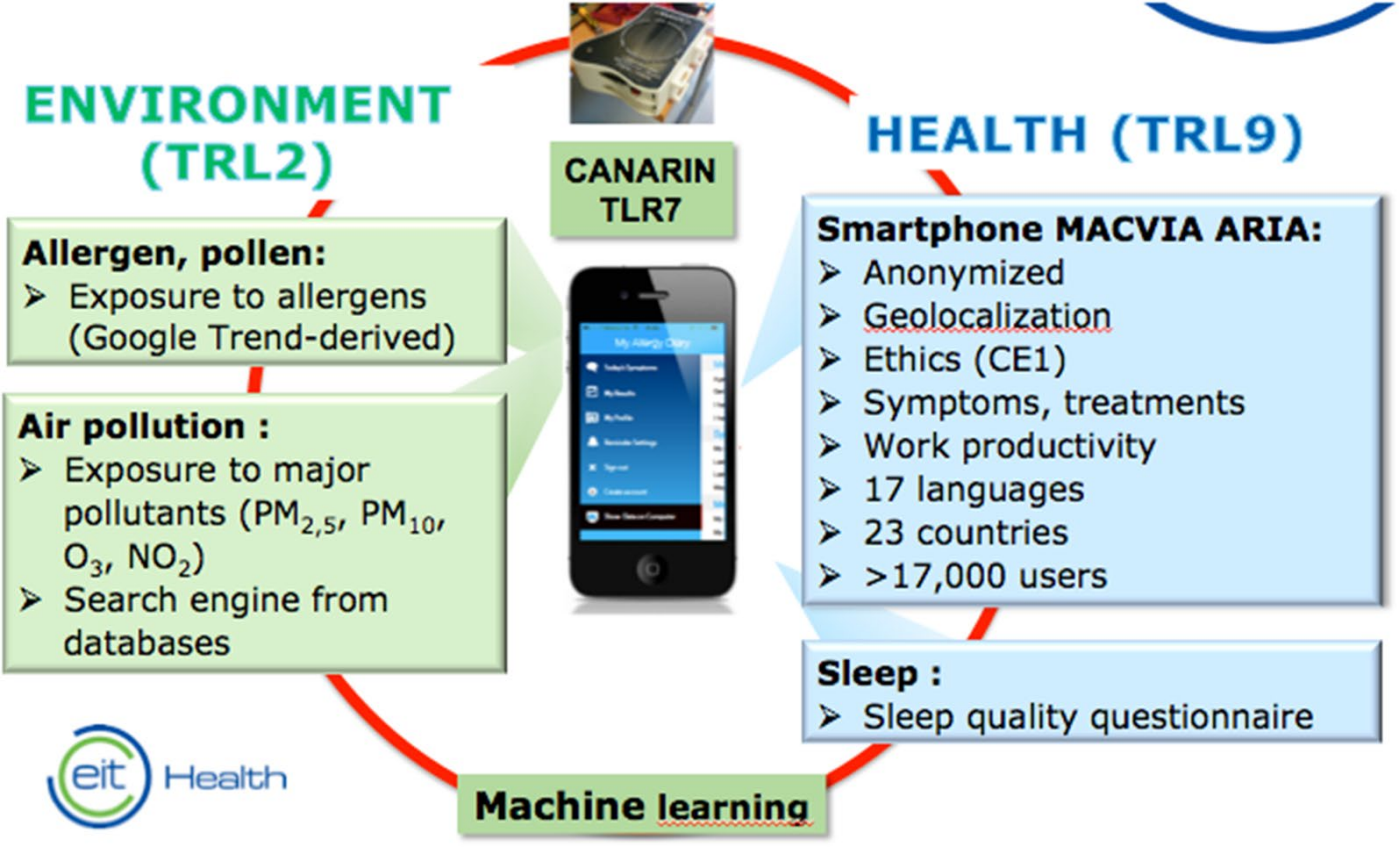
POLLAR m-health tools

The consortium is led by BULL and includes EIT Health members from France and Spain as well as two SMEs (Figs. 2, 3).

- BULL is responsible for the platform. The physician of the hosting platform provider (Santeos) will be responsible for ethical issues, privacy preservation in general and data mining.
- UPMC (Université Pierre et Marie Curie, Paris, France) will provide the personal pollution sampler, medication analyses and the prediction model.
- Grenoble University and Kyomed (France) will be in charge of sleep, data analysis of sleep and patients’ inclusion in year 2. Grenoble University will make innovative capabilities available for complex and big data analysis [60, 61].
- ISGlobal (Global Health Institute, Barcelona, Spain) will be responsible for data analysis.
- AQuAS (*Agencia de Qualitat i Avaluacio Sanitaries de Catalunya*, Barcelona, Spain) will be dedicated to policies.
- Kyomed (SME, Montpellier, France) will provide integrated solutions (Allergy Diary) and business models, as well as support to product design, sales and marketing activities for the project.
- Forum of Living Labs (Paris, France) will prepare and analyze the qualitative data collection of population awareness on air pollution and literacy.
- The National Center of Expertise in Cognitive Stimulation (CEN STIMCO, NGO, Paris, France), as a founding member of the Forum of Living Labs for Health and Independent Living (Forum LLSA, NGO, Paris, France), will prepare and analyze the qualitative data collection of population awareness on air pollution and literacy.
- Neogia (SME, Paris, France) will provide the database of daily trends in air pollution and allergen queries.
- In 2019, the Alfred Health hospital will lead studies in Australia.

### m-Health tools and platform

POLLAR combines TRL2, TLR7 and TLR9 m-health tools (Fig. 3).

#### Allergy Diary

The existing *Allergy Diary* App (Android and iOS) has been tested in 23 countries and 17 languages. The tool has now been deployed to 22 Reference Sites of the European Innovation Partnership on Active and Healthy Ageing (Transfer of Innovation).

**Fig. 4:**
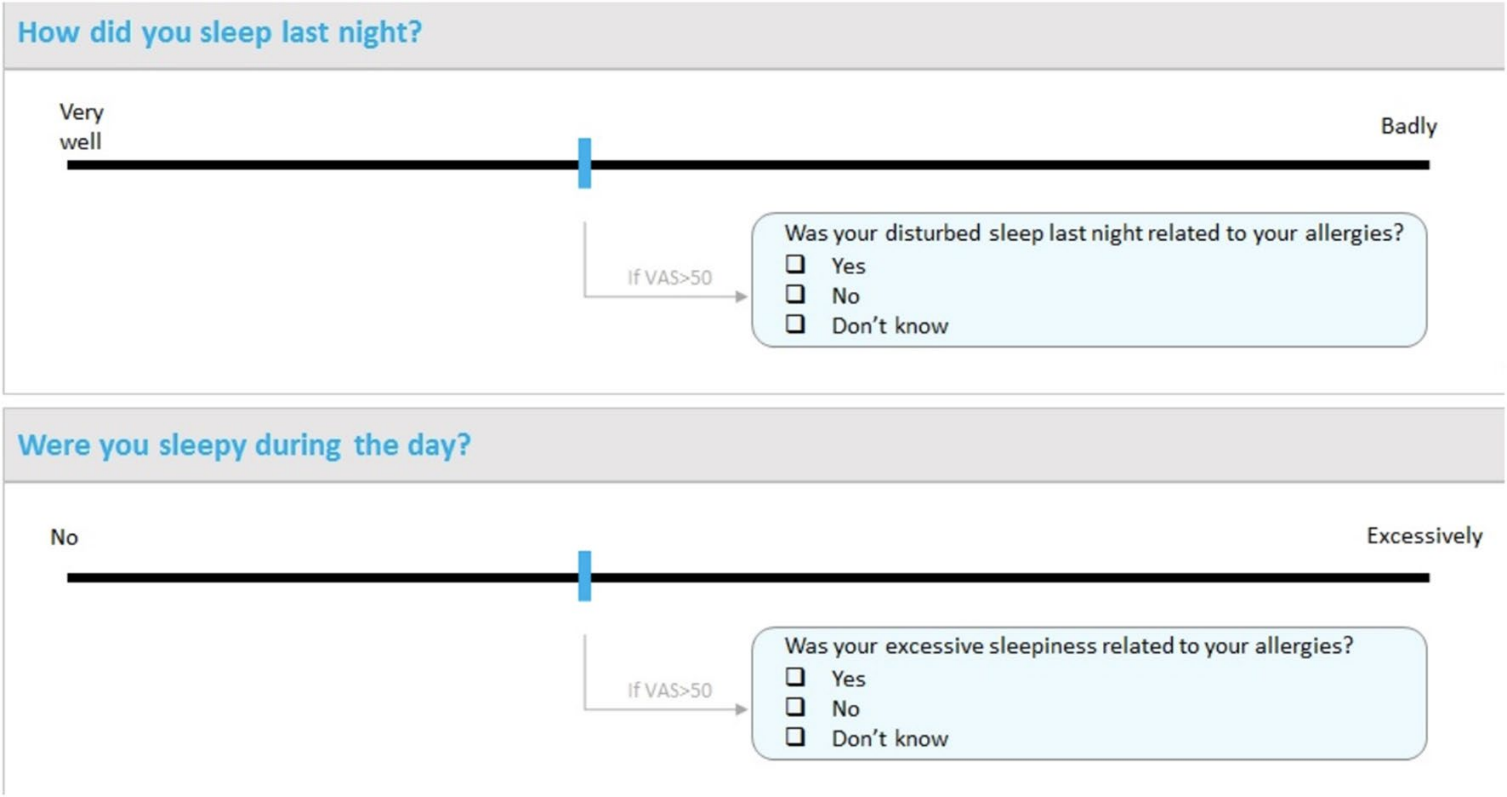
MASK questions on sleep

Sleep is an important component of the social consequences of AR. The *Allergy Diary* has shown that sleep is impaired by some of the components of AR [22, 53]. A new sleep questionnaire is being added to the *Allergy Diary* (Fig. 4).

#### Monitoring of pollen exposure and air pollution

Monitoring of the allergy season will be carried out with an improved Google Trends (GT) method. However, GT has some defects [36, 37], in particular, the lack of quantitative data and the non-exhaustivity of internet. We have developed a new tool derived from GT (TLR2) that will analyze all trends and that will be quantitative for allergen and pollution. A similar method will be used for the assessment of air pollution levels.

#### Set up of the platform, secure storage database and tools for machine and deep learning

The analytic technologies have shown their limits in delivering accurate results and insights. The introduction of artificial intelligence and cognitive computing is bridging the gap. Artificial intelligence and cognitive computing provide technologies and capabilities to solve the challenge of ingesting large amounts of diverse data and to deliver more accurate and timely results and insights.

Thus, main technological challenges focus on delivering a multi-usage and agile cognitive and integrated software suite, leveraging artificial intelligence to enable deep learning capabilities and fast deployments of accurate use cases on multiple environments.

#### Canarin^®^

To assess the individual’s exposure to air pollution in real-time, we will use a remote sensor named CANARIN^®^. The CANARIN^®^ device is intended to provide the user with a cost-efficient means of determining air quality exposure in real-time in the different places the user is located. CANARIN focuses on particulate matter (PM) of 3 sizes (0.1 µm, 2.5 µm and 10 µm). Furthermore, it includes a temperature and humidity sensor as these parameters can affect the performance of the PM sensors. It also allows the geolocalization of the carrier. All the data are stored via WIFI in an ad hoc cloud. CANARIN has been validated.

### Ethical considerations

The Allergy Diary is a CE1 application for which an ethical committee is not necessary. The Terms of Use and Privacy Policy of the App have been reviewed and adapted by lawyers in each of the 23 countries in order to account for differences between countries.

POLLAR will need ethical approval and new regulations in some of the countries (e.g. Loi Jardé in France [62, 63]). We are currently deploying the App to 25 Reference Sites of the EIP on AHA and we have obtained ethical approval from the Köhln-Bohn Region.

The Allergy Diary is completely anonymized except for the geolocation aspect that has been pseudo-anonymized. We have now used k-anonymity [64] to fully anonymize geolocation [65]. We are updating the ethical approval for POLLAR in order to comply with the GDPR [66].

### Test case implementation (Mo 1–12)

We shall use the data from 4 countries with 20,000 users during the pollen season and analyse the interactions between air pollution, sleep and allergens.

1. Data collection

Collection of *Allergy Diary* data over one year during the pollen season (March-July or September in areas with ragweed pollen) and outside the pollen season (September–October in areas without ragweed pollen).

2. Data analysis using the Allergy Diary
  Interactive data analysis during the pollen season.
  Interactive data analysis outside of the pollen season.
  Specific analyses on sleep. We will analyze the trajectories of symptoms reflecting sleep quality and daytime sleepiness along with exposure to air pollution and allergic rhinitis. We will take advantage of the knowledge gained from the two existing cross disciplinary programmes of IDEX Grenoble (Life is made of Choice (https://life.univ-grenoble-alpes.fr/)) and of the Grenoble data institute (https://data-institute.univ-grenoble-alpes.fr). We will use innovative visualization tools for these trajectories that will be included in the Allergy Diary.
  Interactive maps with GIS (geographic information system) technology: GIS is one of many information technologies that have transformed the way geographers conduct research and contribute to society. GIS can be viewed as an integrating technology. With GIS, it is possible to map, model, query and analyze large quantities of data all held together within a single database.
3. Impact of allergy/pollution interactions on prescribed medications in France

Epidemiological studies have suggested a potential causal relationship between air pollution and exacerbations of asthma and allergies. In particular, air pollution exposure is associated with increased medication use and need for rescue medication for asthma and allergies. The potential exaggerating effects of the interaction between pollen and air pollution on asthma and allergic diseases are of serious concern. The collection of data from pharmacy databases for both prescribed and over-the-counter medications for asthma and allergies constitutes an appropriate method for studying the impact of the interaction between air pollution and pollen on asthma and allergy aggravation.

4. Establishment of the business plan

It is expected that POLLAR will generate substantial and highly valuable scientific data as well as information correlating the biological phenomena with the highlighted environmental factors. It is also highly possible that novel technologies or platforms may be developed as a result of the project. Both aspects are not only of scientific but also of commercial value. To capitalize on the value generated both from the data/information and potential novel technology, Kyomed will establish a business plan towards the monetization of these assets. The business plan will include analyses of (1) the properties, functionalities, uniqueness and potential of the data/information or technology generated, (2) the market trends and needs, (3) the competitive landscape; the competitive advantages of the POLLAR offer. It will make further recommendations on the commercial positioning, product placement, pricing and promotional activities for the offer. Financial forecasts and budgeting will also be provided.

5. Education (CEN STIMCO)

Citizens and patients participating in the programme will gain awareness regarding the risk associated with air pollution. This effect will be estimated and results will help disseminate key messages together with the application.

### Test case validation (Mo 12–24)

In year 2, we shall validate the results of year 1 in all EU countries where pollution data are available. We will also provide policies. The test-case implementation will be deployed to account for different climates (allergen exposure) and pollution (low and high levels and different pollutants). We shall use the existing EIP on AHA transfer of innovation network (25 Reference Sites across Europe in 16 languages).

Moreover, Canarin^®^, a personal pollution sampler, will be tested in AR patients with multimorbid sleep disorders (1) to confirm the data obtained using the Allergy Diary and (2) to check the effect of air pollution on sleep and sleep apnea severity [67]. It is well documented that nasal obstruction associated with AR is increasing pharyngeal collapsibility and modulating the severity of moderate to severe sleep apnea. This has been suggested by single night sleep studies and small intervention trails. However, the dynamic of the night after night evolution of OSA severity in relationship with air pollution and AR is poorly documented by repeated objective measurements. The test case validation will address this issue by combining simplified diagnosis methods for assessing the night after night evolution of OSA severity and a synchronization with the Allergy Diary and the Canarin personal pollution sampler. The impact will be huge for patients and the society as some OSA phenotypes might benefit from a better AR management and improve sleep apnea conditions.

## Impact of POLLAR

The innovative aspect of POLLAR lies in the integration of existing hardware and software blocks (BULL) with newly-developed methods in a patient-centric designed set of easy-learning functionalities (Kyomed, CEN STIMCO). These will be embedded in a solution for all stakeholders including patients, clinicians and policy makers. The Allergy Diary represents an innovation that is creating a new market and value network and that will eventually disrupt the existing market and value network.

### Reduction of social and health inequalities

By integrating risk perception analysis and increasing stakeholder engagement, POLLAR aims at (1) bringing more attention to the links between AR and air pollution, (2) educating the public about the threat of air pollution, and (3) efficiently using financial resources to implement a more sustainable solution. POLLAR should reduce health and social inequalities within and between countries, in particular in vulnerable populations (children and old age people).

### Gender dimension

Gender is an important aspect of allergic diseases. Before puberty, there is a male predominance of allergy whereas, after puberty, there is a female predominance [68]. Women may be more susceptible to the effects of air pollution. A specific gender analysis will be carried out in POLLAR to account for gender differences and, if needed, policies will be proposed.

### Economic impact

The *Allergy Diary* can accurately measure loss of work productivity. It is expected that POLLAR will reduce these indirect costs. For industries, the demonstration of the link between AR incidence/severity and productivity underpins the importance of prevention, timely diagnosis, adequate treatment and patient compliance. For public healthcare organizations and private health insurance companies, prevention, timely diagnosis and effective treatments are primordial to healthcare cost management.

### Interactions with EIT Health

#### EIT Health resources needed for POLLAR

EIT Health fosters cross-disciplinal collaborations to tackle major healthcare challenges, such as the growing allergy epidemic and air pollution effects. It provides a privileged frame for validating a comprehensive solution by paving the roads between all stakeholders. It also provides an integrated use of knowledge in medical device development, data management and analytics, and clinical conditions. EIT Health will interconnect POLLAR with its innovation project portfolio and with its CAMPUS (link to “Patient-centred and personalized healthcare description and main outcomes”) and ACCELERATOR programmes, thereby catalyzing both.

#### Relevance of POLLAR for the core mission of EIT Health

The proposed POLLAR solution is aligned with EIT Health core missions: (1) Promote healthy living, lifestyle intervention and self-management of health, (2) Improve healthcare systems, treat and manage chronic diseases and (3) Improve work productivity.

#### Knowledge triangle integration

**Fig. 5:**
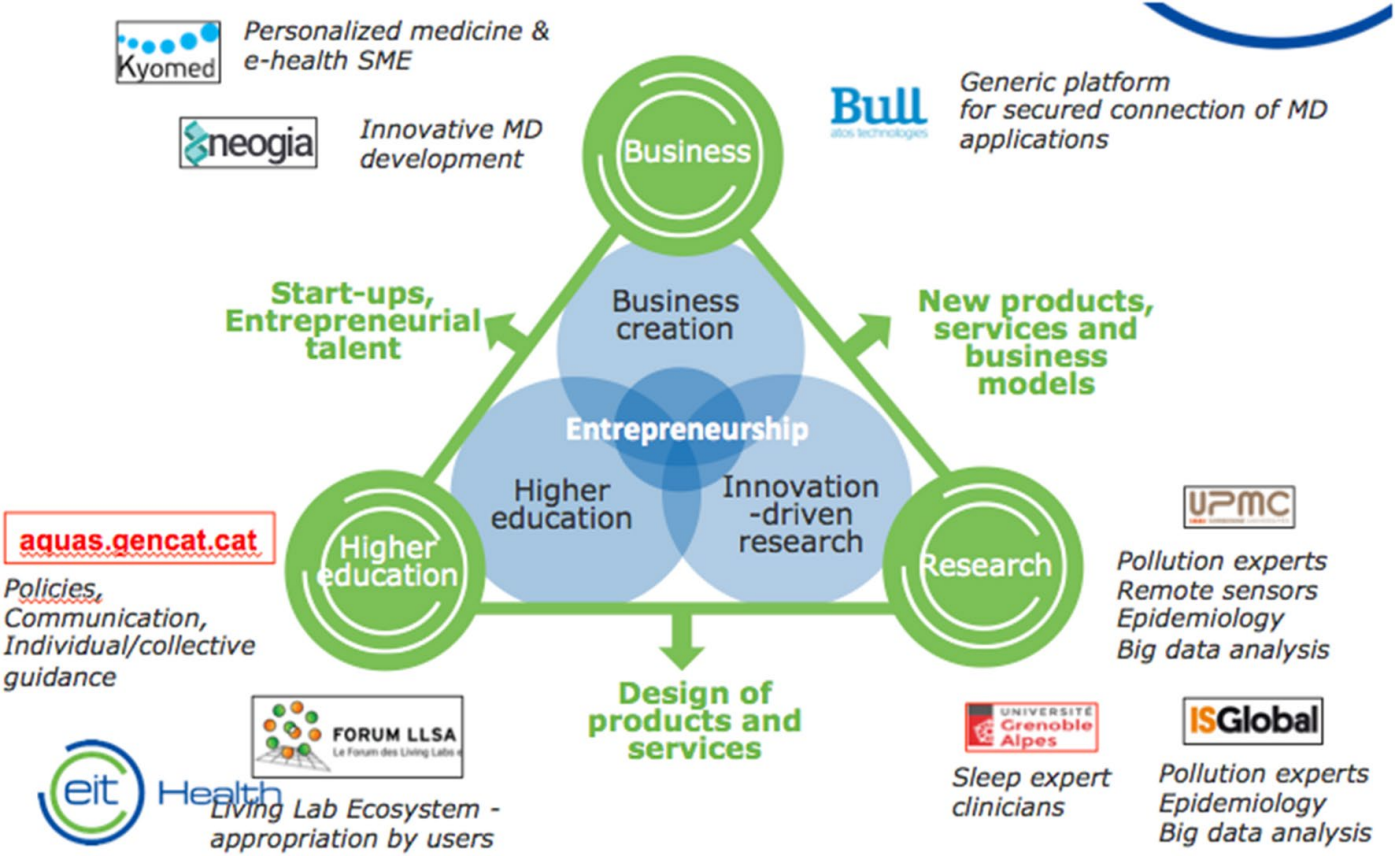
The POLLAR knowledge triangle

POLLAR follows the KIC knowledge triangle closely (Fig. 5).

- *Business and entrepreneurship* POLLAR is typically designing, launching and running a new business, which began as a small business, such as a startup company (Kyomed, Neogia). It is offering a validated product (Allergy Diary, TLR9) which will be embedded with other tools (TLR2) and scaled up. The prevalence of AR and asthma, and the levels of pollution in Europe, represent a huge business opportunity. Being part of the EIP on AHA, goals are an acceleration of time-to-market.
- *Link to accelerator* BULL is a major company which will help to develop new and startup companies (Kyomed, Neogia). It will use the business accelerator effect of the Forum LLSA and will provide services as a catalyst tool for regional and national economic development. It will help to scale the business of several inter-related projects and to catapult promising ideas already working and tested (*Allergy Diary*) onto the market. This will support Europe’s premier innovations that tackle today’s healthcare challenges (air pollution and allergy).
- *Research and technology* The MASK pilot study has shown that guidelines in AR should be revised to account for patients’ self-management which was found to be unexpectedly high [21, 22, 24, 53]. The pilot study suggested several novel pathways for AR treatment. The inclusion of air pollution data is needed to alert patients (Allegy Diary, media, social networks) and to provide new recommendations for a better AR control [45, 69].
  - The patient’s centric approach is essential and will be brought by the CEN STIMCO.
  - A personalized approach targeted to each patient is needed and the *Allergy Diary* can be of great help [70, 71].
  - Sleep is an important component of AR and asthma. It should be better understood and embedded into guidelines.
  - The societal approach is a research project that will bring a novel approach to this complex disease allowing a holistic approach.
  - POLLAR will allow machine learning but also deep learning. The analytical expertise raised in MeDALL (Mechanisms of the Development of ALLergy, a success story of FP7 devoted to systems biology in allergy [57, 72]), with the technologic capabilities of BULL, will make a success story out of POLLAR.
- *Higher education* Higher education will be provided by major teaching groups of Europe (UPMC, Grenoble University and ISGlobal) but also by the Forum LLA and CEN STIMCO. Some modern approaches will be combined with classical education. Integrated training modules are needed. Because of the expected trends in AR, it is of paramount importance to train physicians, other health care professionals, health scientists, lawyers and socio-economic professionals. Transversal training is needed.
- *Health, chronic diseases and society* POLLAR will combine teaching and will cover the relationship between chronic illnesses, chronicity, health and health education, ethics and the assistance relationship, chronicity policies, life with a chronic illness and research methods in the field of human or soft science [73].
- *Management education* The overall care of people suffering from chronic illness requires the coordination of people who will help them throughout life. The *case manager* is a unique correspondent in charge of coordinating care.
- *Patient therapeutic education* will also be provided in collaboration with patients’ organizations (involved in the *Allergy Diary*).

### Benefits for the citizens and the patients

Guidelines have improved the knowledge on rhinitis and made a significant impact on AR management. However, many patients are insufficiently controlled and the costs for society are enormous. Allergic Rhinitis and its Impact on Asthma (ARIA) has evolved from a guideline to care pathways using mobile technology in AR and asthma multimorbidity. ARIA appears to be close to the patient’s needs but real-life data obtained using an App in 22 countries have shown that very few patients use guidelines and that they often self-medicate. Moreover, patients largely use OTC medications dispensed in pharmacies. Self-medication and shared decision making (SDM) centered around the patient should be used more often. The knowledge by patients of peaks of air pollution and allergens will help them to better control their disease. In POLLAR, self-medication strategies and a sentinel network will be integrated in care pathways to optimize the treatment of AR and asthma multimorbidity. These changes should prepare and support individuals, teams and organizations in making organizational change centered around the patient.

### Political agenda

POLLAR is supported by several national and international scientific societies (including EAACI, ERS, IPCRG) and patients’ organizations (EFA and ELF).

One of the POLLAR members is AQuaS (Agencia de Qualitat i Avaluacio Sanitaries de Catalunya, Barcelona, Spain).

The EIP on AHA is involved in POLLAR through the Reference Site Collaborative Network (J Bousquet, M Illario).

POLLAR is a WHO GARD (Global Alliance against Chronic Respiratory Diseases) demonstration project.

## Conclusion

POLLAR aims to propose novel care pathways integrating pollution, sleep and patients’ literacy. It also aims to study the sleep consequences of pollution and its impact on frequent chronic diseases, to improve work productivity, to propose the basis for a sentinel network at the EU level for pollution and allergy, and to assess the societal implications of the interaction.

## Abbreviations

AHA: active and healthy aging
AIRWAYS ICPs: integrated care pathways for airway diseases
AR: allergic rhinitis
ARIA: Allergic Rhinitis and its Impact on Asthma
CDSS: clinical decision support system
CEN STIMCO: Centre d’Expertise National en Stimulation Cognitive
CHRODIS: Joint Action on Chronic Diseases and Promoting Healthy Ageing across the Life Cycle
COPD: chronic obstructive pulmonary disease
DG CONNECT: Directorate General for Communications Networks, Content & Technology
DG Santé: Directorate General for Health and Food Safety
DG: Directorate General
EAACI: European Academy of Allergy and Clinical Immunology
EFA: European Federation of Allergy and Airways Diseases Patients’ Associations
EIP: European Innovation Partnership
EIT Health: European Institute of Innovation and Technology-Health
ELF: European Lung Foundation
EQ-5D: Euroquol
ERS: European Respiratory Society
EU: European Union
EUFOREA: European Forum for Research and Education in Allergy
GARD: WHO Global Alliance against Chronic Respiratory Diseases
GT: Google Trends
HIT: health information technology
ICP: integrated care pathway
ICT: information and communications technology
IT: information technology
JA-CHRODIS: Joint Action on Chronic Diseases and Promoting Healthy Ageing across the Life Cycle
KIC: Knowledge Innovation Community
LLSA: living lab
MACVIA: contre les MAladies Chroniques pour un VIellissement Actif
MASK: Mobile Airways Sentinel NetworK
mHealth: mobile health
OSA: obstructive sleep apnea
POLLAR: Impact of Air POLLution on sleep, Asthma and Rhinitis
SDM: shared decision making
TLR: technology readiness level
VAS: visual analogue scale
